# The evolution of distorted beliefs vs. mistaken choices under asymmetric error costs

**DOI:** 10.1017/ehs.2020.25

**Published:** 2020-05-20

**Authors:** Charles Efferson, Ryan McKay, Ernst Fehr

**Affiliations:** 1 Faculty of Business and Economics, University of Lausanne, Switzerland; 2 Department of Psychology, Royal Holloway, University of London, UK; 3 Department of Economics, University of Zurich, Switzerland

**Keywords:** Cultural evolution, error management theory, herding, social learning, hyperactive agency detection

## Abstract

Why do people sometimes hold unjustified beliefs and make harmful choices? Three hypotheses include (a) contemporary incentives in which some errors cost more than others, (b) cognitive biases evolved to manage ancestral incentives with variation in error costs and (c) social learning based on choice frequencies. With both modelling and a behavioural experiment, we examined all three mechanisms. The model and experiment support the conclusion that contemporary cost asymmetries affect choices by increasing the rate of cheap errors to reduce the rate of expensive errors. Our model shows that a cognitive bias can distort the evolution of beliefs and in turn behaviour. Unless the bias is strong, however, beliefs often evolve in the correct direction. This suggests limitations on how cognitive biases shape choices, which further indicates that detecting the behavioural consequences of biased cognition may sometimes be challenging. Our experiment used a prime intended to activate a bias called ‘hyperactive agency detection’, and the prime had no detectable effect on choices. Finally, both the model and experiment show that frequency-dependent social learning can generate choice dynamics in which some populations converge on widespread errors, but this outcome hinges on the other two mechanisms being neutral with respect to choice.

## Graphical abstract

**Figure:**
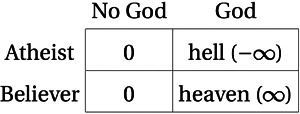

**Media summary:** An evolutionary analysis of distorted beliefs and widespread errors when incentives, cognitive bias, and social learning mix.

## Introduction

In early modern Europe, Christian zealots put thousands of innocent people to death because these innocents were seen to be agents of Satan (Boyer, 2001). In parts of Asia and Africa, ‘penis panics’ have occurred repeatedly. These outbreaks of paranoia centred on the belief that one's genitals were receding into one's body or had been stolen. People resorted to self-destructive measures to protect themselves, and they arbitrarily accused others of genital thievery (Yap, 1965; Sachdev, 1985; Ilechukwu, 1992; Cheng, 1996; Buckle et al., 2007; Bures, 2008). In 2003, the United States justified its invasion of Iraq by arguing that the Iraqi government was manufacturing weapons of mass destruction. In the wake of the invasion, the evidence overwhelmingly indicated that Iraq did not have such weapons, but many US citizens maintained the opposite belief (Gaines et al., 2007).

Whether witches, disappearing genitalia or bombs that do not exist, why do large numbers of people sometimes hold seemingly unjustified beliefs or make seemingly bad choices? Put differently, what is the evolutionary explanation for widespread errors in a risky and uncertain world (Haselton & Nettle, 2006; Foster & Kokko, 2009)? We distinguish between beliefs in error and choices in error. A belief in error means a decision maker holds a belief about her situation that available evidence does not fully justify. A choice in error comes in two forms, ex ante and ex post. From an ex ante perspective, the decision maker makes a sub-optimal choice given the information she has about her situation at the time of her decision. From an ex post perspective, the outcome that follows from a choice is not the best possible outcome given the situation the decision maker actually faces. Under risk and uncertainty, the two perspectives are linked, but not perfectly so. Bad decision making may increase the probability of bad outcomes, but good outcomes are still possible. Analogously, good decision making may increase the probability of good outcomes, but bad outcomes can still occur.

Here, we address errors in belief and errors in choice by examining the effects of three different mechanisms. Each mechanism represents a prominent hypothesis about the origin and persistence of errors. No hypothesis is mutually exclusive with respect to any other, and so we examine the three mechanisms in all combinations.

First, the incentive structure of the current decision-making task can support frequent errors in choice if some errors are costlier than others (Pascal, 1995, originally published in 1670). Under asymmetric error costs, choosing optimally requires the decision maker to minimise expected costs. In turn, people should commit cheap errors with a relatively high probability to reduce the probability of expensive errors. This is simply optimal decision making under risk (McKay & Efferson, 2010). It does not require unjustified beliefs, and choices are errors only from an ex post perspective that conditions on the decision maker's realised environment or situation. Imagine, for example, that Charles offers Ryan a gamble. With probability 0.01, Charles pays Ryan 10,000 dollars. With probability 0.99, Ryan pays Charles one dollar. Objective probabilities are known and verifiable, and so beliefs are perfectly accurate. Ryan accepts the offer, but ex post he typically walks away with one less dollar, a good bet with a bad outcome. The only mechanism of interest is that losing one dollar is far better than losing the opportunity to gain 10,000 dollars. In evolutionary terms, the hypothesis of interest is that evolution has shaped parts of the mind to develop so that they are tolerably adept at general-purpose optimising (Frankenhuis et al., 2013; Barrett, 2015).

Second, error management theory (Haselton & Nettle, 2006; McKay & Efferson, 2010; Johnson et al., 2013; Marshall et al., 2013; Haselton et al., 2015) posits that the incentive structures of ancestral decision-making tasks can support contemporary errors in belief and, by extension, errors in choice. The claim is that many decision-making domains in the ancestral past involved consistent variation in error costs. Cognition for a given domain evolved to process information from the environment in a way that helped ancestral humans avoid especially costly errors. Contemporary humans retain the psychologies in question. If environmental stimuli activate an associated cognitive process, the decision maker chooses in accord with ancestral conditions, and she does so even if the contemporary setting is quite different (Cosmides & Tooby, 2013). Because underlying beliefs are potentially distorted by psychologies adapted to ancestral conditions that may no longer hold, beliefs can be errors, as can attendant choices from both ex ante and ex post perspectives.

Finally, we consider the hypothesis that frequency-dependent social learning supports multiple equilibria and path-dependent cultural evolutionary dynamics (Boyd & Richerson, 1985; Bikhchandani et al., 1992; Young, 1996, 2015; Toyokawa et al., 2019). When people learn socially by observing how common or rare different behaviours are, the path a population takes can depend sensitively on small random events that occur early in the cultural evolutionary process. Consequently, a tradition in which nearly everyone chooses in error, given their situation, can be a stable equilibrium that obtains with positive probability (Boyd & Richerson, 1985; Bikhchandani et al., 1992). This mechanism has been offered as an explanation for the persistence and even origin of many harmful traditions, including foot binding and female genital cutting (Mackie, 1996; Efferson et al., 2015, 2020; Platteau et al., 2018), child marriage (Cloward, 2016) and duelling (Young, 2015).

Importantly, social learning and path-dependent dynamics are perfectly consistent with a rational benchmark in which everyone has Bayesian beliefs and maximises expected payoffs given these beliefs (Bikhchandani et al., 1992). Consequently, the mechanism requires neither unjustified beliefs nor sub-optimal decision making. Nonetheless, social learning could amplify or attenuate the effects of other mechanisms, and so social learning also does not preclude unjustified beliefs and sub-optimal decision making. One of our main tasks here is to examine interactions between social learning and other mechanisms, specifically mechanisms related to contemporary error costs and to psychologies evolved to manage ancestral error costs.

We present a model that combines the three mechanisms above, and we then show results from a behavioural experiment with a design that parallels the model closely. This jointly theoretical and empirical approach has key advantages. It requires us to provide precise operational definitions of the three mechanisms. This is especially critical with respect to contemporary vs. ancestral cost asymmetries as the distinction between these two mechanisms is not always clear (McKay & Efferson, 2010; Marshall et al., 2013). Moreover, our modelling and empirical efforts jointly link the present study to other fields, some theoretical and some empirical. These include expected utility and cumulative prospect theories (von Neumann and Morgenstern, 1944; Kahneman and Tversky, 1979; Tversky & Kahneman, 2000), error management theory (Haselton & Nettle, 2006; McKay & Efferson, 2010; Johnson et al., 2013; Haselton et al., 2015), risk dominance in game theory (Harsanyi & Selten, 1988; Camerer, 2003), information cascades and herding (Bikhchandani et al., 1992; Anderson & Holt, 1997; Chamley, 2004; Goeree et al., 2007; Goeree & Yariv, 2015), and the study of cultural evolution (Cavalli-Sforza & Feldman, 1981; Sperber, 1996; Richerson & Boyd, 2005; Mesoudi, 2011; Henrich, 2015; Morin, 2016; Heyes, 2018). Finally, our joint approach ensures that we have a framework for interpreting our experimental results (Muthukrishna and Henrich, 2019). For example, as explained below, past implementations of the generic experimental paradigm we used (e.g. Anderson & Holt, 1997) led us to expect that social learning would routinely generate path-dependent dynamics and associated harmful traditions in our experiment. The model suggested a more subtle result. Our intuition was wrong; the model was not.

## A model of beliefs and choices with explicit incentives, biased cognition and social learning

The environment takes one of two states, 0 or 1. The ex ante probability of state 1 is *p*_1_ ∈ (0, 1). Individuals have two actions to choose from, which we also label as 0 and 1. Choosing 0 in state 0 is better than choosing 1, and choosing 1 in state 1 is better than choosing 0. Specifically, *u*_00_ is the payoff from choosing 0 in state 0, *u*_10_ is the payoff from choosing 1 in state 0, and *u*_00_ > *u*_10_. Similarly, *u*_11_ is the payoff from choosing 1 in state 1, *u*_01_ is the payoff from choosing 0 in state 1, and *u*_11_ > *u*_01_. This is the ‘explicit incentive structure’ of the decision-making task, which represents the first mechanism we consider.

Two errors are possible. A decision maker can choose 1 when the state is 0, with a loss of *u*_00_ − *u*_10_, or the decision maker can choose 0 when the state is 1, with a loss of *u*_11_ − *u*_01_. If error costs are the same, the explicit incentive structure is ‘symmetric’. If error costs are different, it is ‘asymmetric’. We arbitrarily designate state 1 as the state with relatively large error costs under asymmetric incentives, which means *u*_11_ − *u*_01_ ≥ *u*_00_ − *u*_10_. To illustrate asymmetric incentives, imagine a woman walking through the forest. She spies something long, dark and skinny. Is it a dangerous snake, equivalent to state 1, or just a stick, equivalent to state 0? Treating a snake as a stick is worse than treating a stick as a snake, and so the woman decides to tread carefully. Treading carefully does not require her to process snake-like stimuli in a special way, although we consider this possibility below. Rather, treading carefully simply requires the woman to recognise explicitly that snakes are more dangerous than sticks. A long dark skinny object in the forest is in this sense equivalent to the gamble above that Charles offers to Ryan. Put differently, the explicit incentive structure of a task at hand is a general mechanism that can shape choices via asymmetric costs in ways unrelated to specific decision-making domains and associated selection in the ancestral past.

Our treatment of cognition, in contrast, centres on hypotheses about past selection. Specifically, decision makers do not simply respond to explicit incentives and an ex ante probability; they also learn. Learning may or may not yield beliefs in error depending on the structure of evolved cognition. This is the second mechanism we examine.

Before choosing, each decision maker observes a private signal about the state. She interprets this signal and learns accordingly. Like the state, a signal can take a value of 0 or 1. After observing her signal, a decision maker updates her beliefs about the environment via Bayes’ Rule. Beliefs take the form of a subjective probability that the state is 1. If the decision maker observes 0 as a private signal, her belief goes down. If she observes 1, her belief goes up. Because the signal is private, associated learning is individual. With Bayesian updating, we do not suggest that real people routinely perform Bayesian calculations. Rather, whatever the actual cognitive processes people use, Bayesian beliefs provide the unbiased benchmark. Using Bayes’ Rule in the model thus allows us to posit a precise operational definition of beliefs formed via biased vs. unbiased cognition.

In particular, decision makers interpret private signals in a way that may or may not be accurate. Decision makers think that private signals match the state with probability 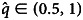. The actual probabilities that govern perceived private signals may be different. If so, decision makers systematically misinterpret signals, and we thus refer to a ‘cognitive bias’. We focus on cognitive biases that distort beliefs in favour of state 1. If the state is 0, the actual probability a decision maker perceives a 0 signal is 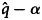 for some 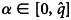. If the state is 1, the actual probability that a decision maker perceives a 1 signal is 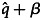 for some 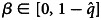. To see why this represents a cognitive bias favouring belief in state 1, consider the extreme case in which 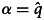 and 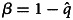. Perceived signals indicate state 1 regardless of the actual state, and thus private signals are completely uninformative. However, because decision makers are unaware of their bias, they interpret perceived signals as evidence for state 1.

An archetypical scenario illustrates. A contemporary human is walking through the forest, and she spies something long, dark, and skinny. Is it a dangerous snake, equivalent to state 1, or just a stick, equivalent to state 0? Ancestral error costs were presumably asymmetric because treating a snake as a stick in the ancestral past was worse than treating a stick as a snake (Haselton & Galperin, 2012). Crucially, however, we considered explicit incentives above as the first of our three mechanisms, and these incentives, whether ancestral or contemporary, are not our concern here. Rather, our concern is the hypothesis that ancestral cost asymmetries led to the evolution of a cognition that distorts contemporary belief formation. Such a cognition would imply that contemporary humans are hypersensitive to snake-like stimuli and draw inferences about the threat of dangerous snakes that are not fully justified by the evidence at hand. Formally, *α*, *β* > 0, and thus beliefs deviate systematically from the unbiased Bayesian benchmark. Many cognitive biases of this sort have been hypothesised owing to ancestral cost asymmetries (Abbey, 1982; Haselton & Nettle, 2006; McKay & Efferson, 2010; Delton et al., 2011; Johnson et al., 2013; Marshall et al., 2013; Haselton et al., 2015; Perilloux & Kurzban, 2015; Zimmermann & Efferson, 2017; Murray et al., 2017), and our experiment below considers a specific bias known as ‘hyperactive agency detection’ (Guthrie et al., 1980; Guthrie, 1993; Barrett, 2000, 2004, 2012; McKay et al., 2018; Maij et al., 2019).

As the final mechanism, decision makers learn socially by observing others. They make choices one at a time in a sequence indexed by *t*. At any given point in the sequence, the decision maker in question has a prior belief that the state is 1, which we call 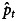. The decision maker observes her private signal and updates her belief. Given the explicit incentive structure and her updated belief, the decision maker has an expected payoff from choosing 0 and an expected payoff from choosing 1. She then makes a choice. Decision makers tend to choose in a way that maximises expected payoffs, but they do not do so with certainty. The parameter *λ* ∈ [0, ∞) controls how strongly choices respond to expected payoffs. For low values of *λ*, choices are relatively noisy and only somewhat responsive to expected payoffs. For high values of *λ*, decision makers are extremely responsive and almost always maximise expected payoffs, which means decision making is relatively systematic.

Social learning occurs because choices are observable. For the first decision maker, at position *t* = 1, her subjective prior is the ex ante objective prior, 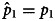. The decision maker observes her private signal, updates her beliefs, and makes a choice observable to everyone else. All downstream decision makers (*t* > 1) update their beliefs after observing the choice at *t* = 1. The ex ante objective prior (*p*_1_), the incentive structure (*u*_00_, *u*_10_, *u*_11_ and *u*_01_), 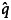 and *λ* are all common knowledge, and so everyone downstream updates beliefs in the same way. The result is 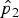, which we treat as a new prior for the decision maker in position *t* = 2. The decision maker at *t* = 2 observes her private signal, updates her beliefs accordingly, and makes an observable choice. Downstream decision makers update their beliefs based on this observable choice, and so the process goes. In sum, the first decision maker only learns individually, while all subsequent decision makers learn both individually and socially. Importantly, if cognition is biased (*α*, *β* > 0), no one is aware of the bias. Decision makers do not account for the bias when updating their beliefs by learning individually via private signals, and they do not account for the bias when learning socially by observing the choices of others.

To avoid any confusion, we would like to explain our use of the word ‘bias’ when referring to social learning. We do not mean biased social learning in the sense of Boyd and Richerson (1985), who used the term ‘biased’ to mean any social learning strategy that generates endogenous cultural evolutionary dynamics. When we say ‘biased’, we mean a cognitive system that processes information in some way that deviates systematically from a Bayesian with an accurate understanding of priors, private information and observed choices. The two views of bias are not at odds; they simply emphasise different questions. Interestingly, recent theoretical research has shown that genetic evolution can support social learning strategies that are both consistent with Bayesian updating and generate endogenous cultural evolutionary dynamics (Perreault et al., 2012; Efferson et al., 2016). Such strategies are biased in the sense of Boyd and Richerson (1985) but unbiased by our definition.

### Model results, analytical

Cost asymmetries in the explicit incentive structure exert a powerful influence on choice by weakening the belief a decision maker requires before choosing 1. Moreover, this mechanism does not require a large asymmetry. Indeed, the largest effects on behaviour occur when moving from no asymmetry to small asymmetries (Supplementary Information). Moreover, the potency of explicit cost asymmetries has nothing to do with the origin of beliefs. Explicit cost asymmetries exert their considerable influence on behaviour regardless of whether beliefs are prior to learning or posterior, and regardless of whether or not beliefs are distorted by cognitive bias. Cost asymmetries mean that decision makers require a relatively weak belief that the state is 1 before choosing 1, and this claim is independent of how beliefs are formed.

Belief formation is a separate process, and we show that beliefs can only evolve in the wrong direction if cognition is biased in a sufficiently strong way (Supplementary Information). Specifically, if cognition is unbiased (*α* = *β* = 0), beliefs evolve in expectation in the right direction. If the state is 0, beliefs that the state is 1 are expected to go down. If the state is 1, beliefs that the state is 1 are expected to go up. If cognition is biased (*α*, *β* > 0), beliefs may or may not evolve in expectation in the right direction. Because we focus on cognitive biases that distort beliefs in favour of state 1, belief evolution when the state is 1 is not especially interesting. The associated cognitive bias (*β* > 0) may speed up the evolution of beliefs in favour of state 1, but it cannot send belief evolution off in the wrong direction. Information from the environment and the cognitive bias point towards the same conclusion.

The interesting scenario centres on the evolution of beliefs in favour of state 1 when the actual state is 0. We show that beliefs evolve in expectation in favour of state 1, and hence in the wrong direction, if and only if 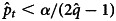. In effect, beliefs are expected to evolve consistently away from reality if and only if decision makers think that private signals are much noisier than these signals really are. For example, if 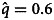, decision makers think that private signals are relatively noisy, *α* can take any value in [0, 0.6], and 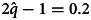. By extension, for any feasible *α* ≥ 0.2, 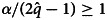. Consequently, 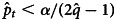 always holds, and thus beliefs favouring state 1 always increase in expectation. In contrast, if 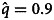, *α* can take any value in [0, 0.1], and 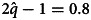. Even if *α* = 0.1, the maximum feasible value, beliefs favouring state 1 only increase in expectation if the prior is sufficiently small, namely if 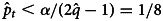. Here, the effects of the cognitive bias are fundamentally limited. The cognitive bias prevents beliefs from converging on the truth, but the bias does not systematically distort belief evolution in the wrong direction. For most priors (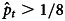), beliefs are expected to move in the right direction, namely towards zero, even though cognition is biased in the other direction.

All in all, our model isolates 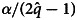 as an important measure of cognitive bias. If 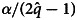 is sufficiently large, beliefs are always expected to evolve in the wrong direction when reality is inconsistent with the bias. Alternatively, if 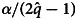 is sufficiently small, beliefs are only expected to evolve in the wrong direction when the decision maker's prior is sufficiently close to the truth. Otherwise, beliefs are distorted by the cognitive bias, but they still evolve in expectation in the right direction.

### Model results, simulation

To provide a complete depiction of how beliefs and behaviour evolve, we developed an agent-based simulation of the exact model detailed above, and we ran simulations under a diverse array of parameter values (Supplementary Information). In particular, we varied the explicit incentive structure (*u*_00_, *u*_10_, *u*_11_ and *u*_01_) and the properties of cognition (*α* and *β*). Like the analysis above, we arbitrarily limit attention to associated biases in favour of 1. This means, if error costs are asymmetric, the asymmetry favours choosing 1 (*u*_11_ − *u*_01_ > *u*_00_ − *u*_10_). We varied the incentive structure to range from no asymmetry to large asymmetries. As explained above, however, the difference between symmetric error costs and small asymmetries is the difference that matters most. In addition, if cognition is biased, the bias distorts beliefs in favour of 1 (*α*, *β* > 0). We specifically set 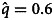 and varied *α* and *β* over the full range of possible values. As explained above, any associated distortions in information processing may or may not be strong enough to lead beliefs to evolve in the wrong direction under state 0.

Finally, we also varied how strongly choices respond to expected payoffs (*λ*), and we varied the ex ante probability of state 1 (*p*_1_). For every combination of parameter values, we simulated 100 independent sequences of 201 decision makers each. Below we provide a link to the files for the simulation, a script for managing the entire project over a user-defined parameter space, and a script for graphing results for each parameter combination.

Here we focus on the interesting case in which state 0 is the most likely state ex ante and the actual state ex post. Specifically, we consider cases in which *p*_1_ = 1/3, and 0 is the actual state, which is expected to happen for 2/3 of all simulated populations. This scenario is interesting for two key reasons. First, if the actual state is 1, cognitive biases and explicit cost asymmetries can only reinforce the evolution of beliefs and choices in a way that is consistent with the actual environment. The tendency for cognitive biases and explicit cost asymmetries to support errors, in contrast, hinges on the environment being in the opposite state, and this is why we focus on cases in which the actual state is 0. Second, by making state 1 ex ante unlikely (*p*_1_ = 1/3), populations that converge on choosing 1 in state 0 are converging on a behavioural tradition that is not just an error, but an error with a relatively high ex ante probability. As explained later, we chose *p*_1_ = 1/3 in our experiment for exactly this reason.

**Figure 1.**
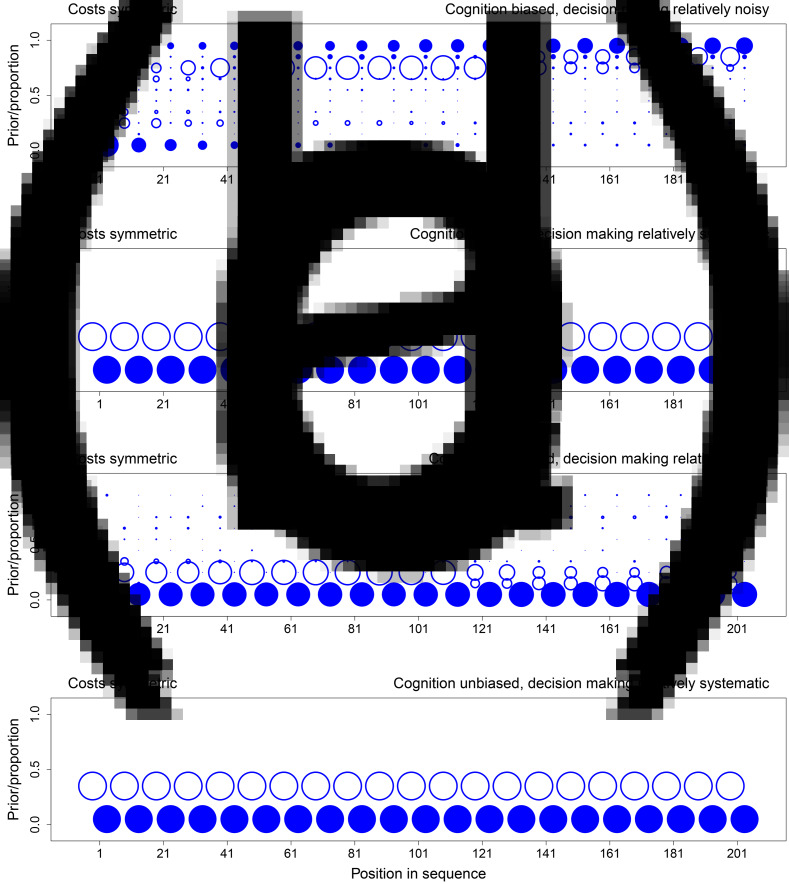
The evolution of beliefs and choices when error costs are symmetric (*u*_11_ = *u*_00_ = 1 and *u*_01_ = *u*_10_ = 0). For each panel, we simulated 100 independent sequences with 1/3 as the ex ante probability of state 1. Each panel shows results for the specific sequences in which the actual state is 0. Over these sequences, the graph shows the distribution of prior beliefs that the state is 1 (open circles) for every tenth decision maker in the sequence. It also shows the associated distribution over the cumulative proportions, by sequence, of decision makers incorrectly guessing state 1 (closed circles). Distributions are represented as bubble plots. (a) Cognition is biased (*α* = 0.5) and decision making relatively noisy (*λ* = 10). (b) Cognition is biased (*α* = 0.5) and decision making relatively systematic (*λ* = 100). (c) Cognition is unbiased (*α* = 0) and decision making relatively noisy (*λ* = 10). (d) Cognition is unbiased (*α* = 0) and decision making relatively systematic (*λ* = 100). See the main text for a detailed description of how to read the graphs and a summary of key results.

**Figure 2.**
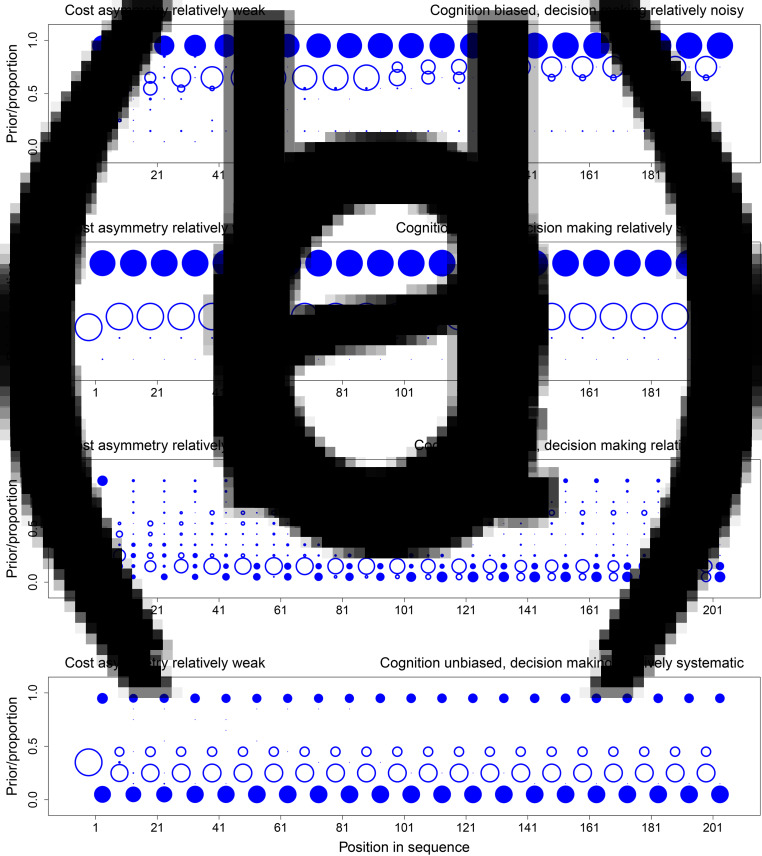
The evolution of beliefs and choices when error costs involve a relatively weak asymmetry (*u*_11_ = 1, *u*_00_ = 0.75, *u*_01_ = 0, and *u*_10_ = 0.25). For each panel, we simulated 100 independent sequences with 1/3 as the ex ante probability of state 1. Each panel shows results for the specific sequences in which the actual state is 0. Over these sequences, the graph shows the distribution of prior beliefs that the state is 1 (open circles) for every tenth decision maker in the sequence. It also shows the associated distribution over the cumulative proportions, by sequence, of decision makers incorrectly guessing state 1 (closed circles). Distributions are represented as bubble plots. (a) Cognition is biased (*α* = 0.5) and decision making relatively noisy (*λ* = 10). (b) Cognition is biased (*α* = 0.5) and decision making relatively systematic (*λ* = 100). (c) Cognition is unbiased (*α* = 0) and decision making relatively noisy (*λ* = 10). (d) Cognition is unbiased (*α* = 0) and decision making relatively systematic (*λ* = 100). See the main text for a detailed description of how to read the graphs and a summary of key results.

**Figure 3.**
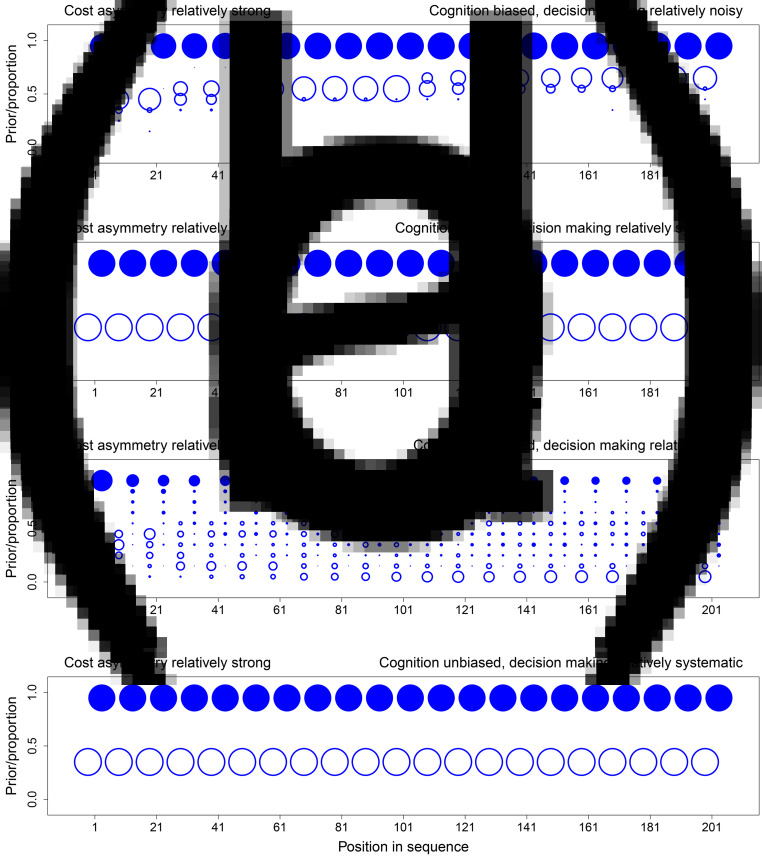
The evolution of beliefs and choices when error costs involve a relatively strong asymmetry (*u*_11_ = 1, *u*_00_ = 0.6, *u*_01_ = 0, and *u*_10_ = 0.4). For each panel, we simulated 100 independent sequences with 1/3 as the ex ante probability of state 1. Each panel shows results for the specific sequences in which the actual state is 0. Over these sequences, the graph shows the distribution of prior beliefs that the state is 1 (open circles) for every tenth decision maker in the sequence. It also shows the associated distribution over the cumulative proportions, by sequence, of decision makers incorrectly guessing state 1 (closed circles). Distributions are represented as bubble plots. (a) Cognition is biased (*α* = 0.5) and decision making relatively noisy (*λ* = 10). (b) Cognition is biased (*α* = 0.5) and decision making relatively systematic (*λ* = 100). (c) Cognition is unbiased (*α* = 0) and decision making relatively noisy (*λ* = 10). (d) Cognition is unbiased (*α* = 0) and decision making relatively systematic (*λ* = 100). See the main text for a detailed description of how to read the graphs and a summary of key results.

Figures 1–3 show the evolution of beliefs and choices under these conditions. We use bubble plots because they allow us to depict the complete distribution of outcomes over all relevant simulations, and the graphs thus provide complete information about simulation results. To read the graphs, take Figure 1a as an example. First consider beliefs. The prior belief for a given decision maker can take values in [0, 1]. Accordingly, we partition this interval into 10 bins, {[0, 0.1], (0.1, 0.2], (0.2, 0.3], …, (0.9, 1]}. For a given position (i.e. a decision maker in the sequence), we calculate the distribution over the prior beliefs of the decision makers in that position, with one decision maker per simulation, and show that distribution as a bubble plot in open blue circles. Bubbles are centred for each of the 10 bins, and the sizes of bubbles are proportional to the frequency of observations for the bin in question. For example, all decision makers in position 1 have the ex ante objective prior as their subjective priors (
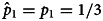
), and so this position has one large open circle at the centre of the interval (0.3, 0.4]. To prevent clutter, we only show the distribution of priors for every 10 positions in the sequence, and distributions are offset slightly to the left relative to the sequence position in question.

Now consider choices. For a given point in the sequence, *t*, we calculate the cumulative proportion of decision makers choosing 1 for each simulated sequence. Let *c*_*n*_ ∈ {0, 1} denote the choice of the decision maker choosing in position *n* for a specific sequence. The cumulative proportion choosing 1 is simply 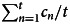. For a given *t*, we have multiple cumulative proportions, one for each simulated sequence. A bubble plot in closed blue circles represents the distribution of these values over sequences. As above, we partition the unit interval into 10 bins and show the distribution of cumulative proportion values over these 10 bins. To illustrate with *t* = 1 in Figure 1a, most simulations under state 0 have cumulative proportions in [0, 0.1], but a few also have cumulative proportions in (0.9, 1]. We show the distribution for every 10 positions in the sequence, and distributions are offset slightly to the right relative to the sequence position in question.

Simulations show four key results.

1. The dynamics of both beliefs and choices unfold slowly when choices respond strongly to expected payoffs and are thus relatively systematic (Figures 1–3, panels b and d, *λ* = 100). To see why, consider the extreme case in which all decision makers always maximise expected payoffs (*λ* → ∞). When this is true, decision makers start off choosing in perfect accord with their private signals. Downstream decision makers can thus infer, with complete accuracy, the private signals of the decision makers in question. Prior beliefs evolve towards one of the two boundaries as the sample of signals and congruent choices grows. Before long, however, prior beliefs become so strong that the weight of history exceeds the informational value of a private signal. At this point, the choice maximising expected payoffs is independent of the signal the decision maker observes (Bikhchandani et al., 1992, 1998). All learning stops because choices no longer reveal private information. For finite but large values of *λ* (Figures 1–3, panels b and d, *λ* = 100), learning never stops in this way (Goeree et al., 2007), but it is slow. It is slow because, even though choices are not independent of signals, probability distributions over choices are nonetheless highly skewed. Observing a choice thus conveys some information but only a little (Cover & Thomas, 2006). With more noise (Figures 1–3, panels a and c, *λ* = 10), probability distributions are less skewed, and observed choices convey more information. This speeds up learning and associated cultural evolutionary dynamics.
2. When explicit error costs are symmetric, beliefs and choices are congruent. If the belief in state 1 is high, choosing 1 is common (Figure 1a). If the belief in state 1 is low, choosing 1 is uncommon (Figure 1b–d). When error costs are asymmetric, beliefs and choices can be incongruent. Choosing 1 can be common even when the belief in state 1 is low (Figures 2b, d and 3b–d).
3. Beliefs only evolve consistently in the wrong direction, namely away from 0, when cognition is biased (Figures 1a, 2a, b and 3a). Here we implement a cognitive bias that we know, given our analytical results above, is a strong bias because 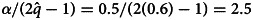.
4. Social learning can generate path-dependent dynamics, but it has no general tendency to do so. Figure 2c, d shows specific situations that support path dependence. Some sequences converge on nearly everyone correctly choosing 0, while other sequences converge on nearly everyone incorrectly choosing 1. For these figures, the cost of an error if the state is 1 is twice the cost of an error if the state is 0. When coupled with the fact that state 1 is half as likely as state 0 (1/3 vs. 2/3), the explicit incentive structure creates no initial bias towards choosing 0 or 1. In addition, cognition is unbiased, and so decision makers do not process information in a systematically distorted way. As a result, both the explicit incentive structure and cognition are neutral with respect to choice. Because incentives and cognition are neutral in this way, the tendency for social learning to generate path-dependent dynamics in behaviour can rise to the surface. In contrast, if explicit incentives are not neutral (Figure 1a–d), or if cognition is biased (Figures 1–3, a and b), sequences do not exhibit this path dependence, and all sequences tend to evolve towards most decision makers choosing either 0 or 1.

## Experimental methods

We conducted behavioural experiments in which subjects made decisions under risk in a shared environment (Supplementary Information). This shared environment took one of two possible states, labelled simply as ‘red’ and ‘blue’. The actual state was not known with certainty, and the basic experimental task was to guess the state correctly. Errors amounted to either guessing blue when the state was red or guessing red when the state was blue. Both errors cost subjects real money. To draw a link with the model above, blue is state 0, and red is state 1.

For a given experimental session, we typically had 34 decision-making subjects and one additional, randomly selected subject who served as the monitor. With probability 1/3 subjects faced the red state, while with probability 2/3 the state was blue. Red was thus ex ante unlikely. Accordingly, a group in which red choices spread under the blue state was a group generating a shared tradition expected to be an error with a relatively high ex ante probability.

In each experimental session we repeated the experiment five times. Treatment conditions were held constant for all repetitions within a session, and each repetition proceeded as follows. The monitor rolled a die to determine the state. A roll of 1 or 2 resulted in the red state, while 3, 4, 5 or 6 resulted in the blue state. The monitor rolled the die while out of sight but within earshot of the other subjects, and all subjects knew the process for determining the state. After rolling the die, the monitor returned to her computer, entered the realised state for the remaining 34 subjects, and the experiment proper began. The experimenter observed all of the monitor's activities to ensure accuracy, and the monitor's payoff did not depend in any way on the realised state or the choices of the other participants.

After the monitor had entered the state, the remaining subjects made their guesses one at a time in a randomly determined order that was independent for each repetition of the experiment. Before making a guess, each subject received a private signal only she could observe. This private signal matched the realised state with probability 0.6, and thus private signals were informative but noisy.

After observing her private signal, each decision-making subject made a guess about the state. Subjects received an increase in payoffs for correct guesses and a decrease for incorrect guesses. As explained below, in some treatments guesses were publicly displayed immediately. As a result, downstream decision makers, in addition to relying on their private signals, could learn socially. In other treatments, guesses were not publicly observable, subjects had only their private signals, and thus learning was strictly individual. Our overall experimental design included eight treatments based on variation in explicit incentives, the presence of a payoff-irrelevant prime intended to induce biased cognition and whether social learning was possible.

### Explicit incentives

In the *symmetric* case, each subject received an endowment of 8 CHF (Swiss Francs). An incorrect guess about the state resulted in a loss of 3 CHF, while a correct guess resulted in a gain of 3 CHF. These gains and losses held regardless of whether the realised state was red or blue. In the *asymmetric* case, endowments were the same, and the losses and gains in the blue state were the same. If the realised state was red, however, a correct guess resulted in a gain of 6 CHF, while an incorrect guess led to a loss of 6 CHF. This means that the cost of an error in the red state was 6 − ( − 6) = 12 CHF, which was twice as much as the error cost of 3 − ( − 3) = 6 CHF when the state was blue.

Our asymmetric treatments involved a weak asymmetry in which the cost of choosing blue when the state was red was only twice the cost of choosing red when the state was blue. Moreover, we exactly offset this asymmetry by making blue twice as likely as red ex ante. The net result was that, in treatments with an asymmetric payoff structure, the a priori expected payoff from choosing red ((1/3)(6) + (2/3)( − 3) = 0) exactly equalled the a priori expected payoff from choosing blue ((1/3)( − 6) + (2/3)(3) = 0). Put differently, before the first subject in a sequence had received her private signal, the cost asymmetry did not produce an initial bias favouring blue or red for risk-neutral subjects. Once subjects started to receive information and learn, this equivalence no longer held.

### Agency prime

Treatments varied in terms of how we described payoffs. In *no agency prime* treatments, the payoff consequences associated with a correct guess were explained in the instructions and on the decision-making screen during the experiment by saying, for example, ‘Your income rises by 3 CHF’. With an incorrect guess, we analogously said, ‘Your income falls by 3 CHF’. This frame was used for both the red and blue states. In *agency prime* treatments, the frame was the same for describing the correct and incorrect guesses under the blue state. However, when describing the payoff consequences under the red state, with the symmetric case as an example, we said, ‘We will reward you with an increase of 3 CHF’ or ‘We will punish you with a reduction of 3 CHF’. For screen shots in the original German, see the Supplementary Information (Figures S3 and S4).

Our aim here was to use a linguistic manipulation to activate the concept of an intentional agent associated with a specific environmental state. The hypothesised cognitive bias of interest is hyperactive agency detection (Guthrie et al., 1980; Guthrie, 1993; Barrett, 2000, 2004, 2012; McKay et al., 2018; Maij et al., 2019). The hypothesis posits that one of the biggest threats ancestral humans faced was other people with furtive, malevolent intentions. Ancestral humans faced two associated errors. They could have assumed an unseen agent was trying to harm them when no such agent was present, or they could have ignored the possibility of an unseen hostile agent when in fact one did exist. The latter error was typically more costly, and this would have increased the tendency to guard preemptively against the hazards of unseen agents. In the end, humans evolved a cognitive bias that discounts the role of chance and overestimates the probability that unseen agents are responsible for many events in life, from the mundane to the extraordinary. Metaphorically, people do not simply see clouds; they see faces in the clouds (Guthrie, 1993).

For our purposes, guessing the state is theoretically equivalent to guessing if an unseen agent is nearby. Under one state, in our case blue, no unseen agent is nearby and costs and benefits simply occur. Under the other state, in our case red, an agent is nearby to discharge costs and benefits as punishments and rewards. This is why we attach the agency prime to the red state. Previous experiments have successfully used both word primes (Shariff & Norenzayan, 2007; Gervais & Norenzayan, 2012) and face primes (Haley & Fessler, 2005; Nettle et al., 2013; Sparks & Barclay, 2013) to activate agency concepts, and linguistic priming effects have been widely documented in behavioural experiments (Tversky & Kahneman, 1981). The question is whether this manipulation shapes choices in a way that is distinct from the effects of explicit material incentives. As explained in our theory section above, such an effect would reflect our operational definition of a cognitive bias.

### Individual and social learning

In *social* treatments, guesses were posted in order, as they occurred, across the top of every subject's screen using either an ‘R’ or a ‘B’. In *asocial* treatments, the character ‘X’ appeared instead, regardless of whether the relevant decision was red or blue.

Overall, our empirical strategy was to design an experiment that captures the potential effects of three mechanisms in all eight combinations. The three mechanisms are asymmetric error costs associated with contemporary explicit incentives, evolved cognitive biases owing to ancestral cost asymmetries and path-dependent cultural evolutionary dynamics. Each mechanism represents a distinct hypothesis about the origin and persistence of errors, and our model suggests that all three should affect if and how decision makers exhibit errors given environmental states.

First, for subjects maximising expected payoffs, treatments including an explicit cost asymmetry should reduce the belief in the red state a subject requires before actually guessing red, which should increase red choices all else equal. Second, treatments using agency language to describe outcomes under the red state should evoke a psychology to mollify the unseen agents who distribute punishments and rewards if the state is red, which should also increase red choices all else equal. Finally, under publicly observable choices, social learning should lead to path-dependent dynamics when other mechanisms are neutral and by extension a subset of populations that converge on a harmful tradition. The neutrality of other mechanisms holds in asymmetric treatments without an agency prime.

We conducted experiments under anonymous laboratory conditions on a local computer network running z-Tree (Fischbacher, 2007) in the Department of Economics at the University of Zurich. We ran sequences of length 34 because this was the maximum length we could implement given the size of the laboratory. Altogether, we ran 20 sessions with 670 subjects, recruited via the laboratory's standard subject pool, for a total of 3365 observations over 100 separate sequences (Supplementary Information). The final sample was 47.5% female with an average age of 22.1 (SD 4.45). Excluding the monitors, subjects made an average of 43.89 CHF in the experiment, and they additionally received 10 CHF each as a show-up fee. Monitors received fixed total payments of 50 CHF. The study was approved by the Human Subjects Committee of the Faculty of Economics, Business Administration, and Information Technology at the University of Zurich. We did not pre-register the study because we collected the data before recent replication studies (Open-Science-Collaboration, 2015; Camerer et al., 2016) and the trend towards pre-registration that followed.

## Experimental results

For all analyses, whether modelling binary or continuous response variables, we rely on ordinary least squares with robust clustered standard errors as a robust approach to estimating average treatment effects under minimal assumptions (Angrist & Pischke, 2009). We conservatively cluster at the session level, which yields 20 clusters. Accordingly, we calculate both the variance-covariance matrix allowing for heteroskedastic errors correlated within clusters (Wooldridge, 2002; Arai, 2011), and we use clustered bootstrapping to obtain 95 and 99% confidence intervals (Cameron & Trivedi, 2005; Angrist & Pischke, 2009). We emphasise results robust to multiple approaches to statistical inference. As additional robustness checks, we also model treatment effects both with and without controls. We provide a link to the data below.

### Red choices

**Figure 4.**
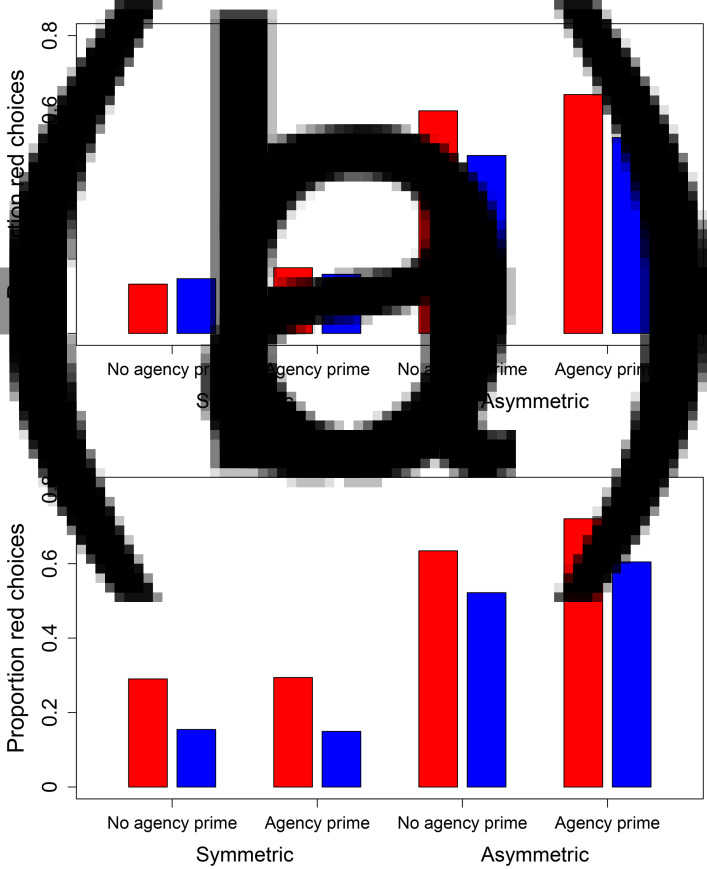
Red choices by treatment and realised state. The proportion of red choices for (a) asocial treatments and (b) for social treatments. The colour of the bars signifies the realised state. Consequently, the red bars represent correct choices in the relatively rare case of a red state, while the blue bars represent errors in the relatively common case of a blue state. These results show that explicit cost asymmetries had an overwhelmingly dominant effect on average choices (see Table 1).

Figure 4 shows the proportion of red guesses conditional on the treatment and the realised state. The figure reveals that explicitly asymmetric error costs had an overwhelmingly dominant effect. Modelling red choices as a function of the treatments confirms this conclusion by showing that asymmetric error costs produced a large and highly significant increase in the rate at which participants guessed red (Table 1, Asym). This means that asymmetric error costs reduced the error rate when the state was red, which was relatively rare, and increased the error rate when the state was blue, which was relatively common. The other treatment dimensions did not robustly affect the rate of red guesses. The results provide some suggestive evidence that the availability of social information slightly increased the probability of participants choosing red, but the effect is not robust to multiple forms of statistical inference (Table 1, Social). In addition, observing a red private signal had a strong and robust positive effect on the probability of making a red choice (Table 1, Signal red).

**Table 1.** Red choices in all treatments. Linear probability models with red choices as the response variable and robust clustered standard errors calculated by clustering on session. In addition, the table shows 95% and 99% confidence intervals calculated with a non-parametric bootstrap clustered at the session level. Independent variables include a dummy for the realised environment for the sequence (Env red), the realised private signal (Signal red), the subject's gender, order in the sequence and treatment dummies (Asym, Agency prime, Social). Columns 2–4 are for models that only include treatments as independent variables. Columns 5–7 add controls.

	Estimate	Cluster boot	Cluster boot	Estimate	Cluster boot	Cluster boot
Parameter	(standard error)	95% CI	99% CI	(standard error)	95% CI	99% CI
Intercept	0.141***	[0.118, 0.312]	[0.074, 0.431]	−0.001	[−0.059, 0.095]	[−0.112, 0.194]
	(0.017)			(0.026)		
Env red				0.043	[−0.022, 0.106]	[−0.040, 0.131]
				(0.032)		
Signal red				0.262***	[0.228, 0.295]	[0.219, 0.306]
				(0.018)		
Female				0.001	[−0.046, 0.049]	[−0.061, 0.065]
				(0.025)		
Order in sequence				−0.0004	[−0.002, 0.001]	[−0.002, 0.002]
				(0.0008)		
Asym	0.409***	[0.191, 0.512]	[0.127, 0.512]	0.393***	[0.252, 0.473]	[0.187, 0.497]
	(0.060)			(0.040)		
Agency	0.026	[−0.211, 0.065]	[−0.297, 0.111]	0.019	[−0.124, 0.083]	[−0.201, 0.135]
	(0.020)			(0.017)		
Social	0.048*	[−0.157, 0.089]	[−0.248, 0.120]	0.070***	[−0.056, 0.107]	[−0.139, 0.179]
	(0.019)			(0.015)		
Asym × Agency prime	−0.027	[−0.153, 0.220]	[−0.176, 0.271]	0.037	[−0.076, 0.199]	[−0.108, 0.249]
	(0.069)			(0.056)		
Asym × Social	−0.024	[−0.179, 0.186]	[−0.231, 0.225]	−0.026	[−0.139, 0.134]	[−0.177, 0.177]
	(0.081)			(0.059)		
Agency prime × Social	−0.047	[−0.117, 0.204]	[−0.137, 0.285]	−0.036	[−0.113, 0.121]	[−0.148, 0.188]
	(0.036)			(0.036)		
Asym × Agency prime × Social	0.125	[−0.096, 0.342]	[−0.154, 0.398]	0.057	[−0.120, 0.248]	[−0.176, 0.301]
	(0.100)			(0.085)		

*(0.05); **(0.01); ***(0.001).

Although the availability of social information did not have a robust effect on the average tendency to choose red, this does not mean that social learning was unimportant. Rather, the result simply means that the possibility to learn socially did not affect average behaviour. For any given social learner, however, the social information available might still have affected her decision making, and before running the experiments we expected social information to influence choices. Indeed, a long tradition of research has shown that people exhibit some tendency to conform when presented with social information about how common or rare different behaviours are (Sherif, 1936; Asch, 1955; Anderson & Holt, 1997; Berns et al., 2005; Morgan et al., 2012; Goeree & Yariv, 2015; Efferson et al., 2016; Muthukrishna et al., 2016; Efferson & Vogt, 2018). We examined such effects by analysing choices in social treatments as a function of frequency-dependent social information. In particular, because our paradigm relies on sequential choices with one choice per subject in a given environment, this analysis avoids the interpretive problems that plague many attempts to identify the causal impact of information about how others behave (Manski, 2000; Angrist, 2014).

Accordingly, Table 2 shows an analysis of red choices in social treatments, where we have added lagged frequency-dependent social information to the independent variables. To specify this variable, let *c*_*n*_ = 1 denote a red choice in position *n* of a sequence and *c*_*n*_ = 0 a blue choice. For any position *t* > 1, lagged social information is the centred proportion of upstream subjects choosing red, 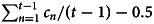. Like the analysis of all treatments (Table 1), the analysis of social treatments shows large and robust effects associated with asymmetric costs and the participant's private signal (Table 2, Asym and Signal red). Asymmetric costs and observing a red private signal both resulted in large and robust increases in the probability a participant chose red. The proportion of observed upstream participants choosing red also had a robust positive effect on the probability of a red choice (Table 2, Lagged social info).

**Table 2.** Red choices in social treatments with frequency-dependent social information. Linear probability models with red choices as the response variable and robust clustered standard errors calculated by clustering on session. In addition, the table shows 95% and 99% confidence intervals calculated with a non-parametric bootstrap clustered at the session level. Independent variables include a dummy for the realised environment for the sequence (Env red), the realised private signal (Signal red), the subject's gender, order in the sequence, the centred cumulative proportion choosing red through the previous period (Lagged social info), and relevant treatment dummies (Asym, Agency prime).

	Estimate	Cluster boot	Cluster boot
Parameter	(standard error)	95% CI	99% CI
Intercept	0.297***	[0.234, 0.344]	[0.208, 0.359]
	(0.025)		
Env red	−0.012	[−0.056, 0.033]	[−0.070, 0.050]
	(0.023)		
Signal red	0.231***	[0.201, 0.257]	[0.191, 0.264]
	(0.015)		
Female	−0.003	[−0.050, 0.040]	[−0.065, 0.053]
	(0.024)		
Order in seq	0.0004	[−0.002, 0.002]	[−0.002, 0.003]
	(0.001)		
Lagged social info	0.701***	[0.612, 0.772]	[0.581, 0.792]
	(0.040)		
Asym	0.125***	[0.073, 0.206]	[0.061, 0.236]
	(0.030)		
Agency prime	−0.025	[−0.055, 0.033]	[−0.066, 0.064]
	(0.016)		
Asym × Agency prime	0.047	[−0.030, 0.120]	[−0.050, 0.138]
	(0.035)		

*(0.05); **(0.01); ***(0.001).

Altogether, our results indicate that frequency-dependent social information affected individual decision making, but it did so without affecting average choices. This result suggests that social learning might have instead affected the variance in choices. Put differently, in comparison to asocial treatments, social treatments might have shifted some of the overall variation in choices from within sequences to between sequences. Such a result would reduce the variation in choices within sequences, and it would be consistent with the hypothesis that social learning supports path-dependent dynamics (Young, 1996, 2015; Bowles, 2004). To test the idea, we now turn to an analysis of the variance in choices by sequence.

### Variance in choices within sequences

Frequency-dependent social learning can generate path-dependent cultural evolutionary dynamics. Path-dependent dynamics, in turn, can generate an important aggregate pattern in which groups differ from one another, but choices within groups are relatively homogeneous (Young, 2015). Whether this aggregate-level pattern occurs, however, can be extremely sensitive to the details of how heterogeneous decision makers respond to social information (Granovetter, 1978; Young, 2009; Efferson et al., 2020). Specifically, conformist social learning at the individual level may or may not translate into path-dependent dynamics at the aggregate level. The most direct route to examining this question is to analyse outcomes directly at the aggregate level (Efferson & Vogt, 2018).

To do so, we treated each sequence of choices as a sample from a Bernoulli distribution and modelled sample variance by sequence as a function of the treatments. Given that our generic experimental paradigm has proven quite conducive to path dependence in the past (Anderson & Holt, 1997), we initially imagined that social sequences would be uniformly more homogeneous than asocial sequences. Our experimental results turned out to be more subtle than this in a way that was consistent with our model. We found that choices were extremely homogeneous, but without path dependence, in asocial sequences with symmetric error costs. For these treatments, blue choices predominated, and adding social information to the mix did not increase homogeneity because choices were already extremely homogeneous. In contrast, social learning led to path-dependent cultural evolution and an associated increase in homogeneity when explicit error costs were asymmetric. As explained below, the incentive structure in asymmetric treatments was relatively neutral with respect to choice, and choices within asocial sequences were correspondingly heterogeneous. Adding social information could then generate path-dependent dynamics and an associated increase in homogeneity.

**Figure 5.**
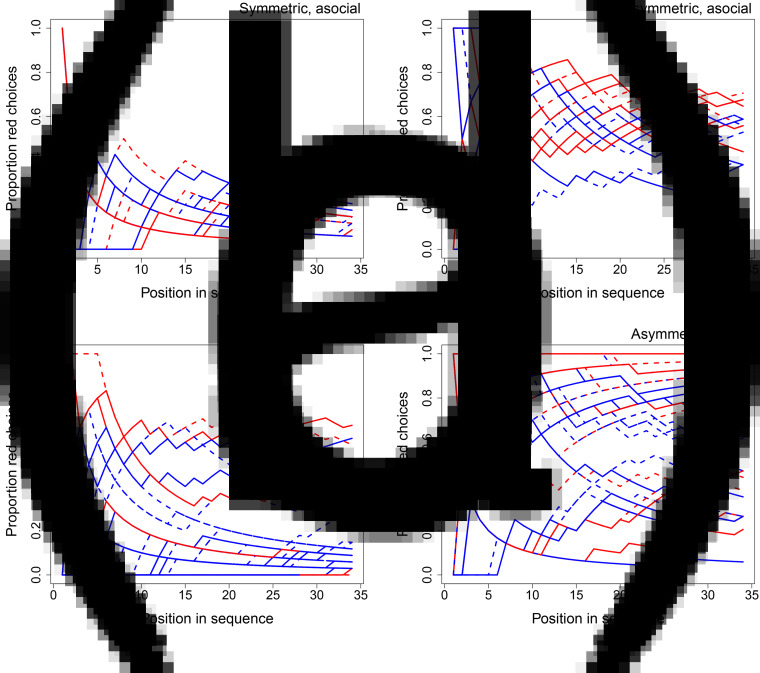
Choice dynamics for all treatments. Let c_*n*_ = 0 denote a blue choice in sequence position *n* and c_*n*_ = 1 a red choice. Given position t, the graphs show the cumulative proportion choosing red by sequence, 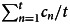, as a function of sequence position, *t*, for all sequences in the experiment. The colour of the line shows the realised state for the sequence in question. Solid lines are for sequences in no agency prime treatments, while dashed lines are for sequences in agency prime treatments. Panels (a) and (b) show asocial treatments, while (c) and (d) show social treatments. Panels (a) and (c) show treatments with symmetric error costs, while (b) and (d) show asymmetric error costs. Social learning led to path-dependent dynamics and an associated increase in homogeneity within sequences when error costs were asymmetric.

Figure 5 shows the choice dynamics for all treatments, and Table 3 shows an analysis of the variance in choices within sequences. In asocial treatments with symmetric costs, the symmetry of error costs did not offset the fact that blue was twice as likely as red ex ante. All sequences converged on blue (Figure 5a), which resulted in homogeneity without path dependence. This left little scope for social information to homogenise choices further (Figure 5c vs. Figure 5a), and regression results show no effect (Table 3, Social and Social × AgencySym).

**Table 3.** Ordinary least squares models with the variance in choices by sequence as the response variable and robust clustered standard errors calculated by clustering on session. The table also shows 95% and 99% confidence intervals calculated with a non-parametric bootstrap clustered at the session level. Independent variables include a dummy for the realised environment by sequence (Env red), a dummy for treatments that allowed social learning (Social), and dummies for the four treatment combinations involving the agency prime and the explicit payoff structure. We used combined dummies for these four combinations in order to avoid three-way interactions. The dummies are defined according to the presence (Agency) or absence (NoAgency) of the agency prime and either symmetric (Sym) or asymmetric (Asym) error costs. Columns 2–4 are for models that only include treatments as independent variables. Columns 5–7 add the sequence-level control.

	Estimate	Cluster boot	Cluster boot	Estimate	Cluster boot	Cluster boot
Parameter	(standard error)	95% CI	99% CI	(standard error)	95% CI	99% CI
Intercept	0.122***	[0.105, 0.183]	[0.106, 0.198]	0.123***	[0.102, 0.183]	[0.097, 0.199]
	(0.012)			(0.012)		
Env red				−0.004	[−0.031, 0.023]	[−0.039, 0.032]
				(0.014)		
Social	−0.019	[−0.080, 0.004]	[−0.090, 0.009]	−0.019	[−0.078, 0.005]	[−0.091, 0.009]
	(0.013)			(0.013)		
AgencySym	0.021	[−0.042, 0.047]	[−0.059, 0.047]	0.021	[−0.043, 0.050]	[−0.061, 0.053]
	(0.014)			(0.015)		
NoAgencyAsym	0.122***	[0.046, 0.143]	[0.036, 0.143]	0.122***	[0.045, 0.143]	[0.035, 0.145]
	(0.013)			(0.014)		
AgencyAsym	0.127***	[0.065, 0.147]	[0.057, 0.147]	0.126***	[0.065, 0.147]	[0.056, 0.150]
	(0.013)			(0.012)		
Social × AgencySym	−0.002	[−0.038, 0.068]	[−0.050, 0.082]	−0.003	[−0.042, 0.069]	[−0.055, 0.085]
	(0.019)			(0.021)		
Social × NoAgencyAsym	−0.066***	[−0.097,−0.005]	[−0.105, 0.006]	−0.066***	[−0.097,−0.006]	[−0.106, 0.005]
	(0.016)			(0.016)		
Social × AgencyAsym	−0.051***	[−0.080,−0.022]	[−0.088,−0.018]	−0.049**	[−0.080,−0.019]	[−0.087,−0.011]
	(0.015)			(0.015)		

*(0.05); **(0.01); ***(0.001).

Adding asymmetric error costs, however, neutralised the tendency for explicit incentives to favour blue choices. Although the blue state was twice as likely as red ex ante, choosing blue in the red state cost twice as much as choosing red in the blue state. Neutralising incentives in this way allowed natural variation in private signals to create considerable variation in choices within asocial sequences (Figure 5b). The result was a highly significant increase in the variance relative to asocial treatments with symmetric error costs (Table 3, NoAgencyAsym and AgencyAsym). Moreover, by pushing choices away from blue, asymmetric error costs increased the scope for social information to homogenise choices within sequences via path-dependent dynamics. This reduction in variance within sequences is exactly what happened (Figures 5d vs. 5b), and interacting the availability of social information with asymmetric costs (Table 3, Social × NoAgencyAsym and Social × AgencyAsym) produced highly significant negative interactions. In sum, asymmetric costs decreased homogeneity within groups, and social information, given asymmetric costs, increased homogeneity within groups.

## Discussion

Both our model and experiment indicate that asymmetries in the explicit incentive structure can exert a powerful effect on choices. Indeed, even without a cognitive bias, rational optimisers with Bayesian beliefs can choose in a way that is optimal under risk, and yet the resulting choices are likely to be costly errors given the state that actually obtains. In effect, smart bets do not always produce good outcomes. More interestingly, explicit contemporary cost asymmetries readily yield scenarios in which smart bets are extremely likely to lead to bad outcomes. We can hijack a staple example from the error management literature to illustrate the point (Haselton & Buss, 2000; Haselton, 2003; Haselton & Nettle, 2006; Johnson et al., 2013; Haselton et al., 2015; Perilloux & Kurzban, 2015; Murray et al., 2017). Imagine a man in a bar approaching every woman there, only to be repeatedly rejected. His persistence could follow from the fact that he is overestimating his chances, but this is not necessary. Perhaps he simply views a missed opportunity as far more costly than a rejection. Such an asymmetry could easily generate the behaviour in question even if the man has an accurate and extremely precise understanding of just how bad his objective chances are (McKay & Efferson, 2010). He persists as a general-purpose optimiser, and he usually goes home alone.

Importantly, cost asymmetries do not need to be especially large, just something other than trivial. Consistent with this idea, we used a relatively small cost asymmetry in our experiment, and it produced a large behavioural effect. The cost of choosing blue when the state was red was only twice the cost of choosing red when blue. Introducing this moderate asymmetry, however, increased the rate of red choices by a factor of nearly 3.5, from 16.9% to 58.8% (Figure 4).

Frequency-dependent social learning can also have dramatic effects via path-dependent dynamics, but the details are decisive. Path dependence implies at least two dynamically stable equilibria. One of the equilibria has most people choosing correctly given the state, while another equilibrium has most people choosing in error given the state. Because widespread errors can be a stable equilibrium, social learning provides a cogent hypothesis about the origin and persistence of seemingly harmful traditions (Mackie, 1996). The existence of path dependence owing to social learning, however, can be hypersensitive to the effects of other mechanisms. Ordinary individual heterogeneity (Granovetter, 1978; Young, 2009; Muthukrishna et al., 2016; Efferson et al., 2020), an evolved combination of individual and social learning (Perreault et al., 2012; Efferson et al., 2016), systematic errors (Goeree et al., 2007) and identity concerns (Efferson et al., 2020) can all destabilise or eliminate equilibria in a system that would otherwise exhibit multiple equilibria, path-dependent dynamics and the potential for stable harmful traditions.

Our results support this overall picture. We found that frequency-dependent social learning strongly influenced cultural evolutionary dynamics, but the details were critical. When error costs were symmetric, the prior distribution over environmental states favoured blue 2/3 to 1/3. Most participants simply chose blue, which left little scope for social learning to amplify random variation in choices early in sequences. In these cases, path dependence played little or no role. Asocial and social learning were similar (Table 3), with perhaps only a small and uncertain tendency for social learning to increase red choices (Table 1 and Figures 5a and c).

When error costs were asymmetric, in contrast, the cost asymmetry favoured red, while the prior distribution over states favoured blue. These two countervailing forces neutralised each other, allowing the aggregate-level effects of social learning to appear. We found that social learning had little effect on average behaviour (Table 1), but it had a large effect on how choices were distributed within vs. between groups. Social learning and associated path dependence shifted much of the behavioural variation from within groups to between groups (Table 3 and Figures 5b, d). Social learning thus transformed errors from a question about individual decision making into a question about the cultural evolution of group traditions. Importantly, we have focussed on frequency-dependent social learning and associated cultural evolutionary dynamics, but this is only one of many possible cultural evolutionary processes (Cavalli-Sforza & Feldman, 1981; Sperber, 1996; Richerson & Boyd, 2005; Mesoudi, 2011; Henrich, 2015; Morin, 2016; Heyes, 2018). Our study does not allow conclusions about the effects of social learning more broadly.

Finally, we found no evidence that our linguistic prime activated a cognitive bias. Error management theory (Haselton & Nettle, 2006; Johnson et al., 2013; Haselton et al., 2015) has provided a rich framework for generating hypotheses and empirical studies about evolved cognitive biases. As discussed above, the key idea is that persistent cost asymmetries in the past shaped the evolution of human cognition to avoid errors that would have been especially costly in ancestral environments. If stimuli activate this ancestral psychology in a contemporary setting, the person in question will choose in a way that avoids the ancestral error. Moreover, she will do so in a way that is somehow distinct from the effects of explicit contemporary information and explicit contemporary incentives.

We have written before about the challenges associated with defining and identifying cognitive biases of this sort (McKay & Efferson, 2010; Haselton et al., 2015; McKay et al., 2018). Notwithstanding the difficulties, at least two empirical strategies exist. First, the researcher can compare two situations that are equivalent in terms of the value of the information available to subjects. One situation is consistent with the hypothesised error management bias, while the other is not. If decision making varies between the two situations in the predicted direction, the result provides support for the cognitive bias. Predicting outcomes in negatively vs. positively autocorrelated sequences (Scheibehenne et al., 2011) provides an example of this approach. Second, the researcher can manipulate payoff-irrelevant stimuli in a way that runs orthogonal to explicit incentives. This is what we have done here. Economic games with eye spots (Haley & Fessler, 2005; Nettle et al., 2013; Sparks & Barclay, 2013; Vogt et al., 2015; Northover et al., 2017) and alternative framings of the Wason task (Cosmides & Tooby, 1992) provide other examples.

In choosing this latter strategy, we varied the frame used to describe outcomes by switching between a neutral frame and a frame that associated unseen agents with a specific state. By varying the frame independently of explicit incentives, our design effectively decomposed asymmetric error costs into a component related to the explicit incentives of the contemporary decision making task and a component related to how cognition evolved owing to ancestral incentive structures. With respect to contemporary incentives, we implemented incentive structures that either did or did not involve explicit error cost asymmetries. With respect to activating biased cognition, our agency prime posited a state (blue) in which costs and benefits simply occur and a state (red) in which unseen agents distribute these costs and benefits as punishments and rewards. We compared this with a control in which costs and benefits for both states were framed in neutral terms. Interestingly, this decomposition mirrors the two dimensions at work when we ask, ‘Does God exist?’ First, the question involves incentives (Pascal, 1995, originally published in 1670). Going to hell instead of heaven is quite costly, while going to church when you could stay home is less so. Second, the question involves beliefs about the origin of outcomes (Barrett, 2012). Under one state, outcomes originate from the dispassionate workings of nature. Under the other state, God is responsible.

Although our prime did not affect choices, this does not mean that people do not have a cognitive bias that overinfers the existence of unseen agents. People may have such a bias, but our agency frame failed to activate it. We cannot exclude this interpretation, and this represents a key limitation of our study. Another recent study, however, also failed to find positive evidence for hyperactive agency detection (Maij et al., 2019).

Regardless, our model suggests that a cognitive bias, even if present and active, may have limited effects in a setting with individual and social learning. To consistently distort the evolution of beliefs away from the truth, the bias must be relatively strong. Otherwise, even if cognition is biased, beliefs can still evolve towards the truth under a wide range of conditions. This implies yet another possibility. Namely, our agency prime may have activated a cognitive bias, but only weakly so, in which case the behavioural effects were too small to detect. Consequently, future research could profit from a more forceful approach to attaching unseen agents to a particular environmental state. For example, some studies have used a paradigm in which participants play a game against partners who are either real people or computers programmed to play like real people (e.g. Falk et al., 2008). This isolates the effects of playing against an active decision maker vs. playing against nature when nature's choices mimic active decision makers. To modify this approach for present purposes, one would require instead that active decision makers, who fill the role of unseen agents, mimic nature.

Finally, even if a cognitive bias does not always lead beliefs to evolve in the wrong direction when the bias conflicts with reality, our model supports the general claim that bias always prevents beliefs from getting arbitrarily close to the truth. This suggests that empiricists can maximise the chances of detecting biases if they work in settings where prior beliefs are close to accurate. We hope this unexpected theoretical finding highlights the value of modelling cognitive biases relative to an explicitly unbiased benchmark (McKay & Efferson, 2010; Zimmermann & Efferson, 2017).

## Supplementary material

For supplementary material accompanying this paper visit http://dx.doi.org/10.1017/ehs.2020.25. click here to view supplementary material

## Research transparency and reproducibility

Code for simulations, raw data, and code for data analysis are available at https://github.com/cmefferson/evoHumSciEffersonetal2020_codeAndData.
