# Supplementary material for: The evolution of distorted beliefs vs. mistaken choices under asymmetric error costs
Source: Evol Hum Sci. 2020 May 20;2:e27. doi: 10.1017/ehs.2020.25 (PMC10427456; doi:10.1017/ehs.2020.25)
Supplement: Supplementary file 1 [file S2513843X20000250sup001.pdf]

Supplementary Information for "The evolution of distorted beliefs versus mistaken choices under asymmetric error costs"

Charles Efferson[1,*], Ryan McKay[2], and Ernst Fehr[3]

[1]Faculty of Business and Economics, University of Lausanne, Switzerland

[2]Department of Psychology, Royal Holloway, University of London, U.K.

[3]Department of Economics, University of Zurich, Switzerland

[*]Address correspondence to *charles.efferson@unil.ch*.

1 A model of beliefs and behaviour with explicit incentives, biased cognition, and social learning

Let the state of the environment be a random variable, X, with support $\{0, 1\}$, and let $P(X = 1) = p_1 \in (0, 1)$. Initially, the environment takes a realised state, x. Subsequently, T individuals get private signals and make observable choices about the state of the environment in a sequence indexed by $t \in \{1, \dots, T\}$. Private signals, S_t, and observable choices, C_t, are also random variables with support $\{0, 1\}$ and respective realisations s_t and c_t. The history at t is the realised sequence of choices through the previous period, $h_t = (c_1, \dots, c_{t-1})$.

Individuals have beliefs about the private signals that are not necessarily accurate[1]. Specifically, individuals believe that their private signals match the realised state of the environment with some probability $\hat{q} \in (1/2, 1)$. The actual probability distribution over private signals, however, may be different. Specifically, for some $\alpha \in [0, \hat{q}]$, $P(S_t = 0 \,|\, X = 0) = \hat{q} - \alpha$. For some $\beta \in [0, 1 - \hat{q}]$, $P(S_t = 1 \,|\, X = 1) = \hat{q} + \beta$. If $\alpha > 0$ or $\beta > 0$, the actual quality of the private signal does not correspond to the perceived quality of the signal, and this will distort

[1]In general, we use hats to identify subjective quantities. When hats are absent, we refer to quantities associated with the actual properties of the decision-making task. Subjective representations do not necessarily match the actual properties of the task.

both individual and social learning. This is how we operationalise the concept of cognitive bias, which we arbitrarily treat as a bias favouring the belief that $X = 1$.

To see the intuition behind our treatment of bias, consider the extreme case in which $\alpha = \hat{q}$ and $\beta = 1 - \hat{q}$. Individuals perceive private signals that indicate $X = 1$ with certainty, and for this reason observed private signals are completely uninformative. Decision makers do not know this because they are not aware of their bias. They process private signals as if these signals provide evidence for $X = 1$. Such inferences are unjustified, however, precisely because, $\forall x \in \{0, 1\}$, $P(S_t = 1 \mid X = x) = 1$.

To specify payoffs, when $C_t = c_t$ given $X = x$, the individual in position t gets a payoff of $u_{c_t x}$. Let $u_{11} - u_{01} \geq u_{00} - u_{10} > 0$. This inequality means that being wrong is always costly, but being wrong when the environment is in state 1 is potentially more costly than being wrong when the environment is in state 0. The decision maker in position t begins with a subjective prior belief, $\hat{p}_t$, that the environment is in state 1. If $t = 1$, assume $\hat{p}_1 = p_1$. Otherwise, the subjective prior depends on the history of observed choices, $\hat{p}_t = \hat{P}(X = 1 \mid h_t)$.

If $\alpha > 0$ when $X = 0$ or $\beta > 0$ when $X = 1$, cognition is biased. For some position $t > 1$, previous individuals in the sequence processed their private signals and made choices based on a distorted understanding of signal quality. The observer at position t has the same distortion, and $\hat{p}_t$ will reflect this fact. More broadly, the decision maker in the first position ($t = 1$) cannot learn socially. She is only an asocial individual learner. If cognition is biased, she processes her private signal in a distorted fashion as a result. Decision makers in all subsequent positions ($t > 1$) learn both individually and socially. If cognition is biased, the bias distorts both individual learning based on private signals and social learning based on observing the choices of others.

If the private signal in position t is $S_t = 1$, call this a "positive" signal. The individual updates her belief using Bayes' Rule and perceived signal quality,

$$\hat{p}_t^+ = \frac{\hat{q}\hat{p}_t}{\hat{q}\hat{p}_t + (1 - \hat{q})(1 - \hat{p}_t)}. \tag{1}$$

If the private signal is $S_t = 0$, the signal is "negative", and updating takes the following form,

$$\hat{p}_t^- = \frac{(1 - \hat{q})\hat{p}_t}{(1 - \hat{q})\hat{p}_t + \hat{q}(1 - \hat{p}_t)}. \tag{2}$$

Choosing c_t after observing private signal s_t yields a subjective expected utility, $\hat{\pi}(c_t; s_t)$. For the four possible combinations of choice and signal, these values are

$$\begin{aligned}
\hat{\pi}_t(0;0) &= \hat{p}_t^- u_{01} + (1 - \hat{p}_t^-)u_{00} \\
\hat{\pi}_t(1;0) &= \hat{p}_t^- u_{11} + (1 - \hat{p}_t^-)u_{10} \\
\hat{\pi}_t(0;1) &= \hat{p}_t^+ u_{01} + (1 - \hat{p}_t^+)u_{00} \\
\hat{\pi}_t(1;1) &= \hat{p}_t^+ u_{11} + (1 - \hat{p}_t^+)u_{10}.
\end{aligned} \tag{3}$$

Subjective expected utilities are converted into a probability distribution over choices using the logit transformation. Specifically, given a logit sensitivity parameter, $\lambda > 0$,

$$\begin{aligned}
P(C_t = 1 \,|\, S_t = 0, h_t) &= \frac{\exp\{\lambda \hat{\pi}_t(1;0)\}}{\exp\{\lambda \hat{\pi}_t(1;0)\} + \exp\{\lambda \hat{\pi}_t(0;0)\}} \\
P(C_t = 1 \,|\, S_t = 1, h_t) &= \frac{\exp\{\lambda \hat{\pi}_t(1;1)\}}{\exp\{\lambda \hat{\pi}_t(1;1)\} + \exp\{\lambda \hat{\pi}_t(0;1)\}}.
\end{aligned} \tag{4}$$

Choice probabilities of this sort (4) mean that decision makers make mistakes with a probability that declines in the expected cost of the mistake. In seemingly paradoxical fashion, such mistakes eliminate equilibria in which cognitively unbiased decision makers ($\alpha, \beta = 0$) converge on incorrect choices given the environment (Goeree *et al.*, 2007). Put simply, mistakes eliminate the possibility that social learning leads to a harmful outcome in the long-run. In theory, this is a limiting result as $T \to \infty$, but Goeree *et al.* (2007) showed that $T = 20$ was typically enough to ensure that experimental participants converged on correct guesses about the state of the environment. For this reason, as we detail below, we used $T = 34$ in our experiments. This was the maximum value we could consistently implement in the lab we used at the University of Zurich.

Explicit cost asymmetries and behavioural biases. To see how state-dependent payoffs affect choice probabilities, let $\bar{P} \in [1/2, 1)$. Further define $z_0 = u_{00} - u_{10}$ and $z_1 = u_{11} - u_{01}$. As explained above, we assume that $z_1 \geq z_0 > 0$, and we now examine how any cost asymmetry affects observable choices. Specifically, what are the conditions required for $P(C_t = 1 \,|\, S_t = 0, h_t) > \bar{P}$? To answer this question, we think of $\bar{P}$ as some behavioural bias in favour of choosing $C_t = 1$, and we examine the conditions necessary for decision making to be at least as biased as the bias represented by $\bar{P}$. Given our assumptions about parameter

values, $P(C_t = 1 \mid S_t = 0, h_t) > \bar{P}$ if and only if,

$$\hat{p}_t > \frac{\hat{q}\Big\{ z_0 - \frac{1}{\lambda}\ln\big((1-\bar{P})/\bar{P}\big) \Big\}}{\hat{q}\Big\{ z_0 - \frac{1}{\lambda}\ln\big((1-\bar{P})/\bar{P}\big) \Big\} + (1-\hat{q})\Big\{ z_1 + \frac{1}{\lambda}\ln\big((1-\bar{P})/\bar{P}\big) \Big\}}. \tag{5}$$

We now treat the right side of (5) as a function, f, of z_1. Again given our assumptions about parameter values, one can readily verify that $f'(z_1) < 0$, and $f''(z_1) > 0$.

Now consider $P(C_t = 1 \mid S_t = 1, h_t)$. As long as,

$$1/2 \leq \bar{P} < \frac{1}{1 + \exp\left\{\frac{-\lambda[(1-\hat{q})z_0 + \hat{q}z_1]}{2\hat{q}-1}\right\}}, \tag{6}$$

then $P(C_t = 1 \mid S_t = 1, h_t) > \bar{P}$ if and only if

$$\hat{p}_t > \frac{(1-\hat{q})\Big\{ z_0 - \frac{1}{\lambda}\ln\big((1-\bar{P})/\bar{P}\big) \Big\}}{(1-\hat{q})\Big\{ z_0 - \frac{1}{\lambda}\ln\big((1-\bar{P})/\bar{P}\big) \Big\} + \hat{q}\Big\{ z_1 + \frac{1}{\lambda}\ln\big((1-\bar{P})/\bar{P}\big) \Big\}}. \tag{7}$$

If $\bar{P}$ is so large that the strict inequality in (6) is not met, then $P(C_t = 1 \mid S_t = 1, h_t) > \bar{P}$ is not possible because of the error structure in the model represented by λ, z_0, and z_1. Let the right side of (7) be a function, g, of z_1, and assume the strict inequality in (6) is satisfied. The requirement that $1/2 \leq \bar{P}$ is, of course, true by assumption. Under our other assumptions about parameter values, one can show that $g'(z_1) < 0$, and $g''(z_1) > 0$.

Intuitively, the signs of the first and second derivatives of f and g tell us the following. Regardless of the private signal, as the cost of being wrong when the environment is in state 1 increases, the subjective prior belief required to generate an observable choice of $C_t = 1$ decreases. Specifically, for some feasible $\bar{P} \geq 1/2$ and a given environmental state $X = x$, the subjective prior needed to generate $P(C_t = 1 \mid X = x, h_t) > \bar{P}$ is a convex decreasing function of z_1. The fact that $f(z_1)$ and $g(z_1)$ decline in a convex fashion is especially notable. One does not need a strong asymmetry (i.e. $z_1 \gg z_0$) to produce a strong behavioural bias towards choices that avoid the more costly error. Most of the effect on observable behaviour occurs when we first introduce a state-dependent asymmetry in the cost of being wrong, and this is why cost asymmetries represent a potentially powerful mechanism. Broadly speaking, explicit cost asymmetries do not necessarily need to be dramatic to generate strong biases in behaviour; they just need to be something other than trivial (McKay and Efferson, 2010).

Cognitive distortions and biased beliefs. To see how cognitive distortions support biased beliefs, we can adopt the perspective of a downstream observer watching the decision maker in position t. Before the decision maker in t receives her private signal, she and the observer have all the same information, and thus they have the same subjective prior, $\hat{p}_t \in (0, 1)$. The decision maker in t, however, is learning individually when she sees her private signal and updates her beliefs as a result. The observer, in contrast, is still a social learner who observes t's final choice and updates her beliefs based solely on public information. Accordingly, from the perspective of the downstream observer, the probability distributions over t's choices conditional on environment take the following form,

$$\begin{aligned}\hat{P}(C_t = 1 \,|\, X = 0, h_t) &= (1 - \hat{q})P(C_t = 1 \,|\, S_t = 1, h_t) \\ &\quad + \hat{q}P(C_t = 1 \,|\, S_t = 0, h_t) \\ \hat{P}(C_t = 1 \,|\, X = 1, h_t) &= \hat{q}P(C_t = 1 \,|\, S_t = 1, h_t) \\ &\quad + (1 - \hat{q})P(C_t = 1 \,|\, S_t = 0, h_t).\end{aligned} \tag{8}$$

The actual probability distributions, however, are

$$\begin{aligned}P(C_t = 1 \,|\, X = 0, h_t) &= (1 - \hat{q} + \alpha)P(C_t = 1 \,|\, S_t = 1, h_t) \\ &\quad + (\hat{q} - \alpha)P(C_t = 1 \,|\, S_t = 0, h_t) \\ P(C_t = 1 \,|\, X = 1, h_t) &= (\hat{q} + \beta)P(C_t = 1 \,|\, S_t = 1, h_t) \\ &\quad + (1 - \hat{q} - \beta)P(C_t = 1 \,|\, S_t = 0, h_t).\end{aligned} \tag{9}$$

Our observer will update her beliefs in one of two ways depending on the choice she observes at position t,

$$\begin{aligned}&\hat{P}(X = 1 \,|\, C_t = 0, h_t) = \\ &\qquad \frac{\hat{P}(C_t = 0 \,|\, X = 1, h_t)\hat{p}_t}{\hat{P}(C_t = 0 \,|\, X = 1, h_t)\hat{p}_t + \hat{P}(C_t = 0 \,|\, X = 0, h_t)(1 - \hat{p}_t)} \\ &\hat{P}(X = 1 \,|\, C_t = 1, h_t) = \\ &\qquad \frac{\hat{P}(C_t = 1 \,|\, X = 1, h_t)\hat{p}_t}{\hat{P}(C_t = 1 \,|\, X = 1, h_t)\hat{p}_t + \hat{P}(C_t = 1 \,|\, X = 0, h_t)(1 - \hat{p}_t)}.\end{aligned} \tag{10}$$

Let $\hat{P}_{t+1}$ be a random variable for the prior public belief in $t + 1$. Assume $X = 0$. When is

the belief that $X = 1$ expected to increase? First note that

$$\begin{aligned} E[\hat{P}_{t+1} \mid X = 0] = P(C_t = 1 \mid X = 0, h_t)\hat{P}(X = 1 \mid C_t = 1, h_t) \\ + P(C_t = 0 \mid X = 0, h_t)\hat{P}(X = 1 \mid C_t = 0, h_t). \end{aligned} \tag{11}$$

After substituting one can show that $E[\hat{P}_{t+1} \mid X = 0] - \hat{p}_t > 0$ if and only if

$$\begin{aligned} -\hat{p}_t \left[\hat{P}(C_t = 1 \mid X = 1, h_t) - \hat{P}(C_t = 1 \mid X = 0, h_t)\right]^2 \\ > -\left[P(C_t = 1 \mid X = 0, h_t) - \hat{P}(C_t = 1 \mid X = 0, h_t)\right] \\ \times \left[\hat{P}(C_t = 1 \mid X = 1, h_t) - \hat{P}(C_t = 1 \mid X = 0, h_t)\right]. \end{aligned} \tag{12}$$

Rearranging yields

$$\hat{p}_t < E[\hat{P}_{t+1} \mid X = 0] \;\Leftrightarrow\; \hat{p}_t < \frac{\alpha}{2\hat{q} - 1}. \tag{13}$$

Condition (13) may or may not be true. If $\alpha = 0$, condition (13) is never true. Equivalently, beliefs cannot evolve away from reality without a cognitive bias. Condition (13) does hold, given $\hat{p}_t$, if α is large enough or $\hat{q}$ is close enough to 1/2. In this case, belief in the proposition that $X = 1$ increases in expectation, and it does so even though $X = 0$.

Importantly, however, this kind of belief evolution does not necessarily occur just because cognition is biased. If $\alpha/(2\hat{q} - 1) \in (0, 1)$, cognition is biased, beliefs move in the wrong direction in expectation if the subjective prior is sufficiently small, but they move in the right direction if the subjective prior is sufficiently large. In such cases, biased cognition has limits in terms of the distortions it can cause. Biased cognition keeps beliefs from getting too close to reality, but it does not consistently lead beliefs to diverge from reality.

Now assume that $X = 1$. In this case,

$$\begin{aligned} E[\hat{P}_{t+1} \mid X = 1] = P(C_t = 1 \mid X = 1, h_t)\hat{P}(X = 1 \mid C_t = 1, h_t) \\ + P(C_t = 0 \mid X = 1, h_t)\hat{P}(X = 1 \mid C_t = 0, h_t). \end{aligned} \tag{14}$$

Substituting allows one to show that $E[\hat{P}_{t+1} \mid X = 1] - \hat{p}_t > 0$ if and only if

$$\begin{aligned} -\hat{p}_t \left[\hat{P}(C_t = 1 \mid X = 1, h_t) - \hat{P}(C_t = 1 \mid X = 0, h_t)\right]^2 \\ > -\left[P(C_t = 1 \mid X = 1, h_t) - \hat{P}(C_t = 1 \mid X = 0, h_t)\right] \\ \times \left[\hat{P}(C_t = 1 \mid X = 1, h_t) - \hat{P}(C_t = 1 \mid X = 0, h_t)\right]. \end{aligned} \tag{15}$$

Rearranging in this case leads to

$$\hat{p}_t < E[\hat{P}_{t+1} \,|\, X = 1] \;\Leftrightarrow\; \hat{p}_t < \frac{2\hat{q} + \beta - 1}{2\hat{q} - 1}, \tag{16}$$

which is necessarily true. This simply tells us that, when $X = 1$ holds, belief in this state increases because cognition, whether biased or not, uniformly supports the belief that $X = 1$.

2 Experimental methods and analyses

All sessions were conducted on a local computer network using z-Tree (Fischbacher, 2007) at the Department of Economics at the University of Zurich. Most subjects were students at the University of Zurich or the Swiss Federal Institute of Technology. Subjects received a show-up fee of 10 CHF. As explained in the main text, each session consisted of five repetitions of the experiment. For each repetition, each subject received an endowment of 8 CHF. Each subject received this money up front in cash. She gained or lost 3 CHF or 6 CHF, depending on the treatment, her guess for the sequence at hand, and the realised state of the environment. In addition to the show-up fee, the monitor received a fixed payment of 40 CHF for the session. Most sessions consisted of 34 decision makers. In two sessions, however, a full complement of subjects did not show up, and so these sessions consisted of 28 (social, symmetric costs) and 33 (asocial, asymmetric costs) decision makers.

Our treatments are most easily summarised and understood by simply viewing the different screens subjects saw. Figures S1 and S2 show the difference between the asocial and social treatments. Figures S3 and S4 show the difference between treatments with no agency prime and with an agency prime. These latter two figures both involve a payoff structure with asymmetric costs. The symmetric case was accomplished by simply replacing “6” with “3”.

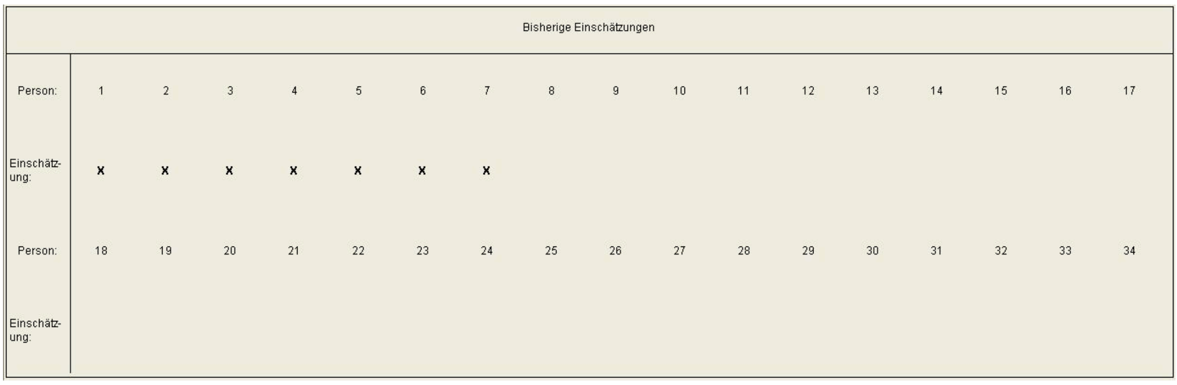

Figure S1. Asocial history. This figure shows the top half of a screen for a hypothetical sequence in progress in an asocial treatment. In this case, seven of the 34 decision makers have already made a guess about the state of the environment. Regardless of which subject was guessing at any given point in time, all subjects saw this type of screen in all periods.

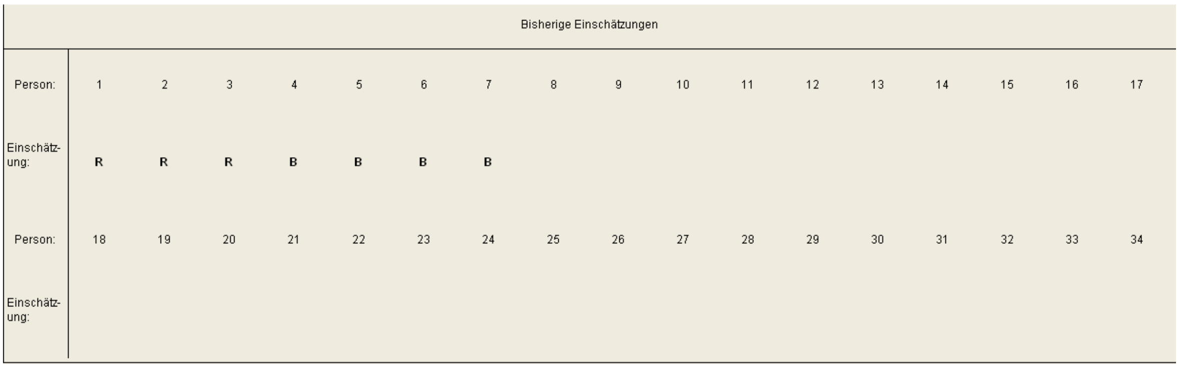

Bisherige Einschätzungen																	
Person:	1	2	3	4	5	6	7	8	9	10	11	12	13	14	15	16	17
Einschätz-ung:	R	R	R	B	B	B	B										
Person:	18	19	20	21	22	23	24	25	26	27	28	29	30	31	32	33	34
Einschätz-ung:																	

Figure S2. Social history. This figure shows the top half of a screen for a hypothetical sequence in progress in a social treatment. In this case, seven of the 34 decision makers have already made a guess about the state of the environment. Regardless of which subject was guessing at any given point in time, all subjects saw this type of screen in all periods.

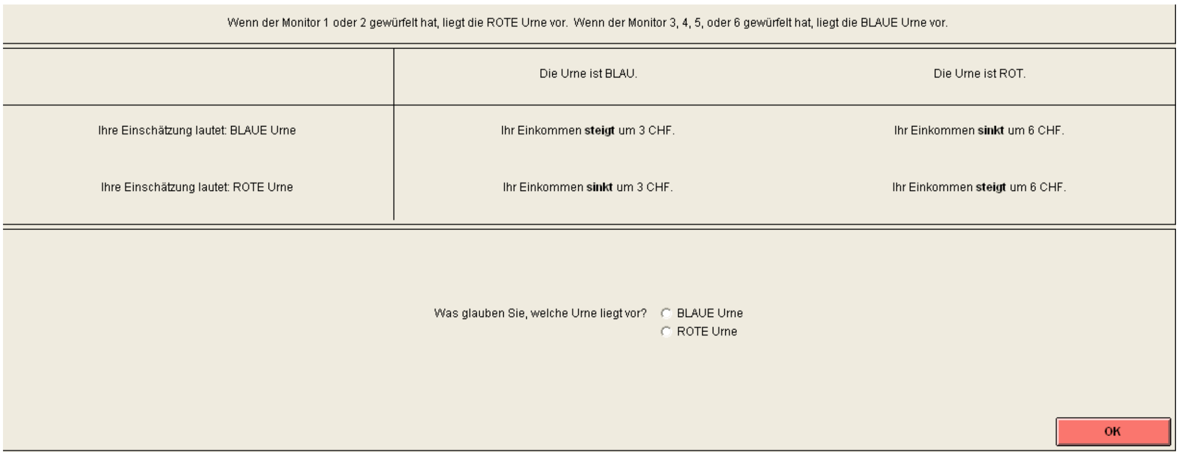

Figure S3. Asymmetric payoff structure, no agency prime. This figure shows a screen shot of the lower half of the screen for a decision-making agent in the treatment with an asymmetric payoff structure and no agency prime. The sentences across the top reminded subjects of the ex ante probability distribution over environmental states. Specifically, these sentences, which read “Wenn der Monitor 1 oder 2 gewürfelt hat, liegt die ROTE Urne vor. Wenn der Monitor 3, 4, 5, oder 6 gewürfelt hat, liegt die BLAUE Urne vor”, translate as “If the monitor rolled 1 or 2, the selected urn is red. If the monitor rolled 3, 4, 5, or 6, the selected urn is blue”. In the payoff matrix below, the phrases “Ihr Einkommen steigt . . .” and “Ihr Einkommen sinkt . . .” are equivalent to “Your income rises . . . ” and “Your income falls . . . ” respectively. In the symmetric case, the values under “Die Urne ist ROT” (“The urn is red”) would have simply been 3 instead of 6. Finally, the subject makes a guess by responding to the question, “What do you think, which urn was selected,” which is a translation of the question pictured above that reads, “Was glauben Sie, welche Urne liegt vor?” Depending on the treatment, the top half of the screen would have shown either an asocial history (Figure S1) or a social history (Figure S2).

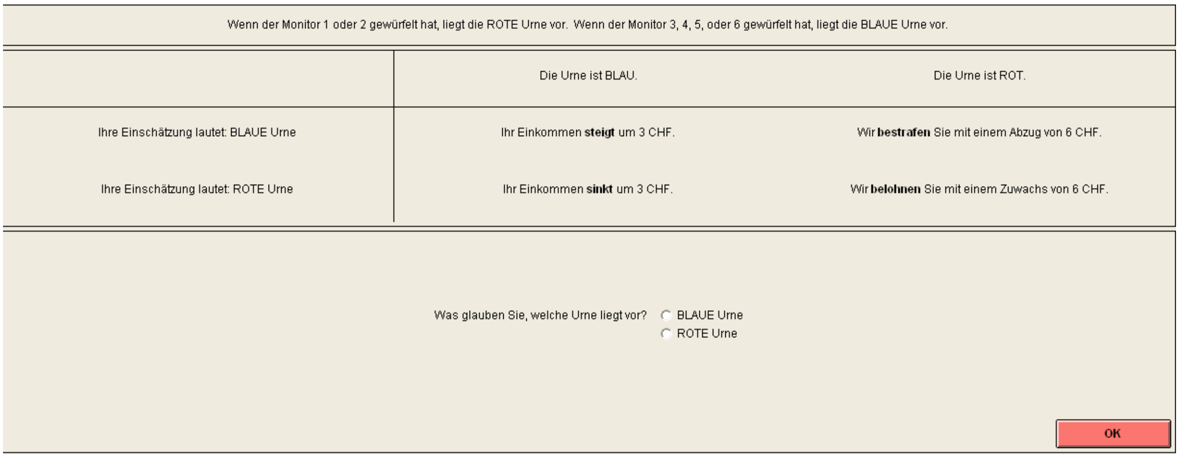

Figure S4. Asymmetric payoff structure, agency prime. This figure shows a screen shot of the lower half of the screen for a decision-making agent in the treatment with an asymmetric payoff structure and agency priming. The sentences across the top reminded subjects of the ex ante probability distribution over environmental states. Specifically, these sentences, which read "Wenn der Monitor 1 oder 2 gewürfelt hat, liegt die ROTE Urne vor. Wenn der Monitor 3, 4, 5, oder 6 gewürfelt hat, liegt die BLAUE Urne vor", translate as "If the monitor rolled 1 or 2, the selected urn is red. If the monitor rolled 3, 4, 5, or 6, the selected urn is blue". In the payoff matrix below, the phrases "Wir bestrafen Sie mit einem Abzug . . ." and "Wir belohnen Sie mie einem Zuwachs . . ." are equivalent to "We punish you with a deduction . . ." and "We reward you with an increase . . ." respectively. In the symmetric case, the values under "Die Urne ist ROT" ("The urn is red") would have simply been 3 instead of 6. Finally, the subject makes a guess by responding to the question, "What do you think, which urn was selected," which is a translation of the question pictured above that reads, "Was glauben Sie, welche Urne liegt vor?" Depending on the treatment, the top half of the screen would have shown either an asocial history (Figure S1) or a social history (Figure S2).

References

Fischbacher, U. (2007). z-Tree: Zurich toolbox for ready-made economic experiments. *Experimental Economics*, **10**(2), 171–178.

Goeree, J. K., Palfrey, T. R., Rogers, B. W., and McKelvey, R. D. (2007). Self-correcting information cascades. *Review of Economic Studies*, **74**, 733–762.

McKay, R. and Efferson, C. (2010). The subtleties of error management. *Evolution and Human Behavior*, **31**(5), 309 – 319.
